# Interaction of Hazelnut-Derived Polyphenols with Biodegradable Film Matrix: Structural, Barrier, and Functional Properties

**DOI:** 10.3390/foods15010107

**Published:** 2025-12-30

**Authors:** Ilayda Hızır-Kadı, Evren Demircan, Beraat Özçelik

**Affiliations:** 1 Department of Food Engineering, Faculty of Chemical and Metallurgical Engineering, Istanbul Technical University, 34469 Istanbul, Türkiye; 2 Department of Food Engineering, Faculty of Engineering, Izmir Institute of Technology, 35430 Izmir, Türkiye

**Keywords:** hazelnut by-products, polyphenols, biodegradable films

## Abstract

The study presents a sustainable approach to valorizing hazelnut processing by-products, specifically skins and shells, through their conversion into bioactive polyphenol-rich extracts using pressurized hot water extraction (PHWE), an environmentally friendly green technology. PHWE yielded extracts with total phenolic contents of 25.4 mg GAE/g dw (shell) and 83.7 mg GAE/g dw (skin), which were incorporated into biodegradable poly(vinyl alcohol)/carboxymethyl cellulose (PVA/CMC) films at concentrations of 1–3% (*w*/*v*). The resulting composites were comprehensively characterized in terms of structural, mechanical, thermal, and barrier properties. FTIR, DSC, and XRD analyses demonstrated strong hydrogen bonding, increased thermal stability, and reduced crystallinity due to polyphenol–polymer interactions. Phenolic incorporation enhanced UV-blocking capability, increased antioxidant activity by up to five-fold, and reduced oxygen permeability from 0.048 to 0.015 (cm^3^·mm·m^−2^·day^−1^·atm^−1^) (69% reduction, *p* < 0.05), compared to neat PVA while maintaining desirable transparency (>70%). Optimal formulations (HSkE-II) exhibited a 39% increase in elongation at break and improved flexibility without compromising film integrity. Application tests using fresh-cut apples, watermelon, and chicken revealed significant reductions in microbial growth (up to ~1.2 log CFU/g), lipid oxidation, and weight loss during storage, confirming the films’ potential for active food packaging. This work highlights an efficient valorization strategy for nut industry by-products and demonstrates their functional integration into sustainable biodegradable packaging systems.

## 1. Introduction

Food loss and waste (FLW) is widely recognized as an urgent global challenge due to its profound environmental, economic, and social consequences. According to the FAO [1], considerable quantities of FLW are generated at every stage of the food supply chain, from primary production and processing to distribution and final consumption, demonstrating the need for effective strategies to transition toward a more sustainable and resilient food system. One promising approach involves the valorization of agro-industrial by-products, which not only reduces waste but also enables the recovery of high-value bioactive compounds that can be incorporated into the food sector [2].

The hazelnut (*Corylus avellana* L.) is a member of the birch family (*Betulaceae*) and is classed as a shelled fruit that grows on trees. Türkiye is widely recognized as the world’s leading producer of hazelnuts and represents the primary source of global hazelnut production [3,4]. During the processing of hazelnuts, a variety of waste materials are generated, including hazelnut shells (HSh), hazelnut skins (HSk), along with green husks and tree leaves. These by-products contain structurally diverse phenolic compounds including catechin, gallic acid, quercetin, protocatechuic acid, syringic acid, and procyanidins which possess well-documented antioxidant and antimicrobial activities [5,6,7,8,9]. The presence of phenolic compounds, which exhibit potent antioxidant properties, in HSh and HSk, signifies that the evaluation of these by-products can contribute to the development of more sustainable food systems [10]. Importantly, no previous study has systematically integrated pressurized hot water extraction (PHWE) derived hazelnut shell and skin polyphenols into PVA/CMC matrices and evaluated their structure and function relationships through comprehensive physicochemical and food application tests. This gap presents an opportunity for both sustainable waste valorization and the development of high-performing bioactive packaging materials.

The pervasive utilization of synthetic food packaging materials, such as plastics, poses considerable environmental challenges, including water, air and soil pollution, primarily due to their non-biodegradable nature. Noteworthy progress has been achieved in the domain of biodegradable plastics, derived from renewable natural resources, to develop biodegradable materials that demonstrate comparable functionality to oil-based polymers [11]. Among these materials, PVA has attracted considerable attention due to its biodegradability, non-toxicity, excellent film-forming ability, and compatibility with natural polymers. Although PVA is a synthetic polymer primarily derived from petrochemical sources, it is well known for its biodegradability under appropriate environmental conditions. To further enhance its performance and sustainability profile, PVA is commonly blended with natural polysaccharides such as carboxymethyl cellulose (CMC), a renewable cellulose derivative. When blended with CMC, the resulting films exhibit improved compatibility, intermolecular hydrogen bonding, and biodegradability. Consequently, PVA/CMC composite films represent a relevant material platform that directly addresses plastic-related environmental concerns while meeting key functional requirements (e.g., oxygen barrier properties, biodegradability) [12,13]. CMC further contributes by enhancing water retention, structural reinforcement, and functional group availability for interactions with phenolics. Previous studies have shown that incorporating natural extracts into PVA-based films can improve antioxidant capacity, UV-blocking efficiency, or antimicrobial activity [12,13,14]. However, these improvements are often system-specific, and the molecular mechanisms governing polymer–phenolic interactions remain insufficiently detailed in the literature.

Film enhancement through phenolic incorporation is governed by intermolecular forces such as hydrogen bonding and alterations in polymer chain mobility. These interactions can modulate crystallinity, thermal transitions, oxygen diffusion pathways, and mechanical performance. Despite existing studies on vegetable tannins, essential oils, or fruit extracts, hazelnut-derived phenolics possess a different structural profile characterized by high flavanol and phenolic acid content that may produce distinct effects on polymer network organization, yet this has not been systematically investigated [13,15]. Furthermore, the industrial relevance of hazelnut by-products, coupled with the sustainability advantages of PHWE, underscores the novelty and importance of a detailed evaluation of these materials in active packaging applications.

The primary objective of this study is to valorize hazelnut by-products, specifically shells and skins, into functional biodegradable packaging materials. To this end, these by-products were incorporated into PVA/CMC-based films, and the resulting structural, mechanical, and barrier properties were systematically evaluated. PVA and CMC were selected as the film matrix, while hazelnut shell (HShE) and skin (HSkE) extracts, obtained via pressurized hot water extraction (a green and sustainable method), were employed as natural bioactive additives. These phenolic compounds were incorporated into the film matrix at concentrations of 1%, 2%, and 3% (*w*/*v*), and films were fabricated using the solvent-casting method. The present study focused on exploring the molecular interactions between phenolics and polymers through FTIR spectroscopy and thermal transitions (DSC), alongside evaluating their impact on barrier, mechanical, and optical properties. Moreover, the shelf-life studies were conducted using fresh watermelon, sliced apple, and chicken. The films were subjected to a seven-day test period to evaluate their efficacy in preserving the quality of the fruit, and a 20-day test period was conducted to assess their impact on lipid oxidation and antibacterial activity in chicken. The use of pressurized hot water extraction a green, environmentally friendly technique and the evaluation of the films on food products from different categories during storage are among the most distinctive aspects of this study compared to existing literature.

## 2. Materials and Methods

### 2.1. Materials and Reagents

Hazelnut skin and shell belonging to the Çakıldak variety were supplied by a local company from Ordu, Türkiye. CMC (MW: 250 kDa, food grade; DS = 1.2) and PVA (99% hydrolyzed; MW: 85–124 kDa) were purchased from Merck KgaA, Darmstadt, Germany. Other chemical compounds and reagents were analytical grade and procured from Merck KgaA, Germany. The fresh fruits and freshly packaged chicken breast meats were obtained from the local market and utilized in a storage performance evaluation of the developed films.

### 2.2. Extraction of Polyphenols from Hazelnut Shell (HSh) and Skin (HSk)

The HSh and HSk were rinsed with tap water, air-dried and then oven-dried (Memmert HPP110, Schwabach, Germany) at 60 °C for 24 h to ensure the thorough elimination of residual moisture [16]. The dried samples were milled using a knife mill (Sinbo-SCM2934, Istanbul, Türkiye) and sieved to a mesh size of less than 0.5 mm [17]. The powders were maintained at 4 °C in hermetically sealed containers to reduce oxidation and moisture uptake. The extraction of HSh and HSk was performed in a 500 mL steel extraction vessel, with the PHWE (Separex SF100, Champigneulles, France) at 150 °C and 1.4 MPa for 60 min, following the method reported by Kasapoğlu et al. [18] with minor modifications. The extracts were filtered through Whatman No. 1 paper before being stored in glass bottles at 4 °C until analysis

### 2.3. Characterization of Hazelnut Shell (HShE) and Hazelnut Skin Extract (HSkE)

#### 2.3.1. Total Phenolic Content (TPC)

The total phenolic content of HShE and HSkE was identified based on the method applied by Mertdinc et al. [18]. Briefly, 1.5 mL of Folin–Ciocalteu reagent and 1.2 mL of 7.5% (*w*/*v*) sodium carbonate (Na_2_CO_3_) solution were incorporated into 200 μL of the extracts. After keeping at 25 °C for 30 min in the dark, absorbance was measured on a UV-Vis spectrophotometer (BioTek, HT, Winooski, VT, USA) at 765 nm. The results were reported as mg gallic acid equivalents per g of dry weight (mg GAE/g dw). All samples were subjected to analysis in triplicate.

#### 2.3.2. Total Antioxidant Activity (TAA)

To identify total antioxidant capacity, a CUPRAC (copper (II) reducing antioxidant capacity) assay was employed. Briefly, 100 μL extract samples were stirred with 1 mL of ammonium acetate buffer (pH = 7), 1 mL of 7.5 × 10^−3^ M neocuproine solution prepared with 100% ethanol, 1 mL of 10^−2^ mM copper (II) chloride solution, and 1 mL of distilled water, respectively. The mixtures were maintained in the dark for 30 min and absorbance was read at 450 nm (BioTek, HT, USA) and results were expressed in mg Trolox equivalent/100 g sample. All samples were analyzed in triplicate [19].

#### 2.3.3. High-Pressure Liquid Chromatography (HPLC)

The HPLC analysis was performed utilizing a Shimadzu 20A series liquid chromatography apparatus, which was coupled with an autosampler, degasser, column oven, and a SPD M20A model PDA detector (Shimadzu Corporation, Kyoto, Japan). The chromatographic separations were conducted on an ACE C18 column (150 mm × 4.6 mm, 3 μm). A gradient elution was employed, utilizing a mobile phase comprising water with 0.75% formic acid (mobile phase A) and methanol with 0.75% formic acid (mobile phase B). The flow rate was set at 1 mL/min, the injection volume was 10 μL, and the temperature of column was maintained at 40 °C. A 55 min gradient program was employed, with the gradient profile outlined below: 0–3 min: 5% B, 3–18 min: 40% B, 18–45 min: 80% B, 48–50 min: 100% B, 52–55 min: 5% B.

### 2.4. Fabrication of Biodegradable Composite Films

**Figure 1:**
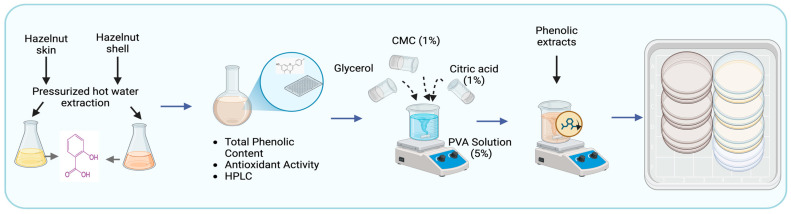
Schematic preparation of PVA-based biodegradable films containing phenolic extracts from hazelnut skin and shell.

The solution casting method was employed to fabricate the biodegradable composite film, using PVA as the polymer matrix. PVA (5% *w*/*v*) was dissolved in distilled water and heated at 70 °C for 1 h. Then, 9 mL of the PVA solution was used as the film-forming base. Subsequently, 1 mL of CMC (1% *w*/*v*), 350 μL of glycerol as a plasticizer and 850 μL of citric acid (CA) (1% *w*/*v*) were mixed into the warm PVA solution [20]. Different concentrations were prepared for the PVA-HShE and PVA-HSkE composite films (1, 2 and 3% *w*/*v* PVA). Three replicates were prepared for each concentration. Samples were coded with control (neat PVA), HShE-I (1%), HShE-II (2%), HShE-III (3%) and HSkE-I (1%), HSkE-II (2%), HSkE-III (3%). These mixtures were then poured onto a clean, smooth glass plate and left to dry overnight at room temperature (50 ± 5% relative humidity). The films were dried for approximately 24 h until a constant weight was achieved, which was taken as the drying endpoint. A schematic diagram of the preparation of the composite film is shown in Figure 1.

### 2.5. Film Properties

#### 2.5.1. Structural Properties (FTIR, XRD)

FTIR spectra of the biodegradable film samples were recorded using a PerkinElmer FTIR spectrometer (PerkinElmer, Waltham, MA, USA) in the range of 4500–400 cm^−1^ with a resolution of 4 cm^−1^, using 32 scans per sample [21].

The samples’ diffraction diagrams were measured between 10° and 50° using a Bruker D8 Advanced X-ray diffractometer from Germany that generated CuKα radiation. These samples were then exposed to an X-ray beam (40 kV, 30 mA) at a rate of 2°/min [22].

#### 2.5.2. Thermal Properties (DSC)

DSC analysis was carried out to check the variation in thermal properties of HShE -HSkE films and neat PVA by a differential scanning calorimeter (TA Q100 Instruments, New Castle, DE, USA). Approximately 5–8 mg of film sample was placed in an aluminum hermetic pan. Samples were then heated from 20 °C to 500 °C at a range of 20 °C/min under nitrogen with a flow rate of 50 mL/min

#### 2.5.3. Optical Properties

The colorimetric properties of the films were determined using a Konica Minolta colorimeter (Konica Minolta CR-400, Tokyo, Japan). The opacity of the prepared films was determined using a UV-visible spectrophotometer (BioTek HT, USA). The spectra were measured at wavelengths between 200 and 700 nm. Each type of film was measured three times, and the average value was computed [23]

#### 2.5.4. Mechanical Properties

Mechanical properties of the films, including tensile strength (TS) and elongation at break (EAB), were measured using a universal testing machine (TA PLUS, Ametek, Berwyn, PA, USA). Film samples (2 cm × 2 cm) were mounted and stretched using extension grips at a rate of 50 mm/min. The thickness of each film was measured using a digital micrometer with a precision of 0.001 mm (HITEC 207-01, Beijing, China) [20].

#### 2.5.5. Barrier Properties (Water Vapor Permeability (WVP), Oxygen Permeability (OP))

WVP of the films was measured according to ASTM E96-00 [24] with slight modifications [25]. The film samples were placed over a circle of 8 cm diameter test tubes including 15 g of anhydrous CaCl_2_ (100% RH, 23 °C) and each sample was weighed. The weight of test tubes was measured at 24 h intervals for 7 days. A graph of the change in weight (g) versus time (h) was plotted and the slope was used to calculate the WVTR (water vapor transmission rate g/m^2^h) using Equations (1) and (2).

(1) $$WVTR=\frac{The\;graph\;slope}{A}$$

(2) $$WVP=\frac{WVTR}{\Delta P}XT$$

T is the composite film thickness (mm), A is the exposed area (m^2^) and ΔP (kPa) is the difference in water vapor pressure between the inside of the cup (100% RH) and the surrounding environment (50% RH) at 23 °C. WVTR is the water vapor transmission rate (g/m^2^h), the exposed area of the test cup was 0.0028 m^2^.

The oxygen permeability (OP) of the film was assessed by measuring the gas transmission rate (GTR) at 50% relative humidity (RH) using a gas permeability tester (Brugger Feinmechanik, GDP-C, Freiburg im Breisgau, Germany) in accordance with the standard method outlined in ASTM D3985-95 [26]. The film was positioned between the upper and lower test vessels. The system was then subjected to a process of vacuuming, after which oxygen gas was introduced into the upper vessel. Pressure changes in the upper vessel were recorded at varying time intervals. The oxygen transmission rate (OTR) (cm^3^ m^−2^ day^−1^) was determined, and the OP (cm^3^ mm^−2^ day^−1^ atm^−1^) of the film was calculated using the following equation (Equation (3))

(3) $$OP=\frac{OTR}{\Delta P}\times T$$

T is the thickness of the film and ΔP is the pressure difference between two chambers (atm).

#### 2.5.6. Antioxidant Activity (AA) of Films

##### DPPH (2,2-Diphenyl-1-picrylhydrazyl)

The antioxidant activity of the film samples was initially determined by means of the DPPH (2,2-diphenyl-1-picrylhydrazyl free radical scavenging) assay, as described by Jridi et al. [27], with slight modifications. The results were expressed as mg Trolox equivalent per 100 g of sample. The analysis of all samples was conducted in triplicate.

##### CUPRAC (Copper (II) Reducing Antioxidant Capacity)

The CUPRAC assay, a methodology originally described by Apak et al. [19], was conducted to evaluate the antioxidant capacity of films. The results were expressed as mg Trolox equivalent per 100 g sample. All samples were analyzed in triplicate.

#### 2.5.7. Biodegradation Analysis

The biodegradability of the film samples was examined by means of a soil burial method according to ASTM D5988-12 [28] with minor changes. A quantity of 100 g of soil was placed into a beaker and then mechanically mixed with 150 mL of water. The mixture was left for a period of 2 h to allow water to penetrate the soil. The dried film samples (3 × 3 cm^2^) were embedded in this soil and water mixture for 28 days. The beakers were incubated at 27 °C, with water added periodically to compensate for evaporation. After removing soil adhering to the film surface, the film samples were removed at regular intervals and dried at 50 °C. The percentage biodegradation of the films was then calculated using the equation (Equation (4)):

(4) $$\%Degradation\;of\;films=\frac{\left(Wo-Wd\right)\times 100}{Wo}$$

W_0_ is the initial dry weight of the film sample and Wd is the dry weight of the film after the biodegradation test period.

### 2.6. Applications of the Films on Fresh Fruits and Chicken

The prepared PVA films were employed for the purpose of covering the test subjects, which comprised fresh watermelon, fresh-cut apple and chicken. The fruits and chicken were procured from the same local market in Istanbul on the same day. All fruit samples were sourced from a single batch, and their ripeness was selected to be moderate, based on acceptable visual and color criteria. For each food type and film formulation, three independent parallel samples were prepared for each sampling day. The samples were randomly divided into equal groups and stored in a separate glass container (internal volume = 150 mL), which was sealed with each film, leaving a large air headspace around the sample during storage. At each sampling day, a different container was randomly selected and opened for analysis to avoid repeated handling of the same sample. The films were cut to 5 × 5 cm to ensure full surface coverage of the top of the containers. All samples, the prepared film samples (HShE-I, HShE-II, HShE-III, HSkE-I, HSkE-II, HSkE-III), neat PVA and conventional stretch film were stored in the refrigerator at 4 ± 1 °C.

#### 2.6.1. Appearance

The quality parameters of the stored fruit pieces were recorded on the first day and after 7 days of storage and the chicken samples were recorded on days 0, 5, 10, 15 and 20 of storage. Following the designated period, alterations in the samples’ color, juiciness and appearance were meticulously recorded. The decay progression of the fruits was subject to close observation, thorough photographic documentation and evidence was obtained using a digital camera.

#### 2.6.2. Weight Loss

The percentage of weight loss was calculated using the following equation (Equation (5)). Wt represents the weight of the samples at recording day, and Wi represents the initial weight of the samples.

(5) $$\%Weight\;loss\;rate=\frac{\left(Wi-Wt\right)\times 100}{Wi}$$

#### 2.6.3. Antibacterial Activity (AbA) of Composite Films

The AbA examination was conducted on days 0, 5, 10, 15, and 20 of storage for chicken. To assess the antibacterial activity of the nine coated films against Salmonella typhimurium, the method outlined by Ahmed et al. [29] was employed with slight modifications. This assay was then repeated on three separate occasions. The expression of microbiological data is presented as a logarithm of colony-forming units per gram (log CFU/g).

#### 2.6.4. Lipid Oxidation

The lipid oxidation assessment was conducted on days 0, 5, 10, 15, and 20 of storage for the chicken samples. The peroxide value of the chicken samples was determined according to Dhakal et al. [30] with some modifications. All analyses were conducted in triplicate, and the results were expressed as meqV/100 g sample.

### 2.7. Statistical Analysis

All analyses were carried out in triplicate. The results were subjected to one-way analysis of variance (ANOVA) using SPSS software (IBM SPSS Statistics, version 28, Armonk, NY, USA). For storage experiments, parallel packages were prepared for each formulation, and a different package was opened and analyzed at each sampling day. One-way ANOVA was applied to compare treatments within each experimental condition, and Tukey’s post hoc test was used to determine significant differences between means (*p* < 0.05). Results are presented as mean ± standard deviation (SD)

## 3. Results

### 3.1. Total Phenolic Content (TPC)

The TPC values of HShE and HSkE are shown in Table 1. HShE exhibited a notably lower TPC (25.44 mg GAE/g dw) than HSkE (83.18 mg GAE/g dw). This outcome is consistent with previous findings reporting that hazelnut skins generally contain higher concentrations of extractable phenolics, particularly flavanols and phenolic acids, compared with hazelnut shells [31].

The TPC of HShE obtained in the present study aligns well with the values reported by Herrera et al. [8], who applied a similar green extraction technique and obtained TPC values ranging between 31.37 and 41.75 mg GAE/g dw. Variability among studies is expected, as PHWE selectivity depends strongly on solvent polarity, temperature, extraction time, and biomass characteristics. Earlier research has also shown that hazelnut shell phenolic yields can be considerably lower when using less efficient extraction methods such as conventional solvent extraction, ultrasound, or low-temperature water extraction, with values reported as low as 3.5–12 mg GAE/g dw [32]. These discrepancies highlight the importance of extraction parameters, particularly particle size, which Yuan et al. [33] demonstrated to significantly influence extraction efficiency, with smaller particle sizes yielding higher TPC. This effect can be attributed to the increased specific surface area and reduced diffusion path length associated with finer particles, which facilitate solvent penetration into the lignocellulosic matrix and enhance mass transfer of bound phenolic compounds. Moreover, smaller particle sizes promote more effective disruption of cell wall structures, thereby improving the release of phenolic compounds during hydrothermal extraction processes [34]. Comparable studies investigating green or eco-friendly extraction techniques have reported a broad range of total phenolic contents for hazelnut by-products, largely depending on the extraction method and raw material. Elmas et al. [35] demonstrated the effectiveness of microwave-assisted, ultrasound-assisted, and pressurized liquid extraction for antioxidant recovery from hazelnut skin and meal. Similarly, Capaldi et al. [16] reported substantially higher phenolic contents for hazelnut skin (176.3–325.7 mg GAE/g dw) using intensified green extraction strategies. In contrast, studies focusing on hazelnut shell and employing conventional or less intensive extraction methods have reported considerably lower phenolic yields (3–9 mg GAE/g dw), highlighting the strong influence of both extraction conditions and biomass type on phenolic recovery [33,36]. Reported TPC values in the literature vary widely depending on extraction methodology; therefore, comparisons are discussed qualitatively.

In contrast, hazelnut skins typically contain substantially higher extractable phenolic levels due to their richer composition of catechin, gallic acid, quercetin derivatives, and procyanidins. This explains the markedly higher TPC observed for HSkE in this study. Previous literature has reported wide variations in hazelnut skin TPC, largely attributable to methodological differences in extraction temperature, solvent polarity, and hydrothermal severity [37,38,39]. In summary, the TPC analysis verified that both HShE and HSkE contain a wide array of polyphenols, with hazelnut skins presenting a significantly richer phenolic profile. These findings support the potential of hazelnut by-product extracts as promising natural antioxidants for incorporation into bioactive food packaging films.

### 3.2. Total Antioxidant Activity (TAA)

The TAA values of HShE and HSkE was calculated according to the CUPRAC assay (Table 1). HSkE exhibited a notably higher antioxidant capacity (638.47 mg TEAC/g dw) compared with HShE (331.23 mg TEAC/g dw), reflecting the well-established differences in phenolic composition between the two hazelnut by-products. Hazelnut skins are known to contain notably higher levels of flavanols, phenolic acids, and procyanidins, which contribute strongly to reducing power in CUPRAC assays.

Comparable trends have been reported in previous studies evaluating the antioxidant properties of hazelnut by-products using CUPRAC and DPPH methods [10,33,40]. These studies consistently showed that hazelnut skin extracts possess antioxidant activities four to five times greater than those of shell extracts, attributable to their richer polyphenolic profile. For instance, Alasalvar et al. [10] reported a DPPH scavenging activity of ~78% for hazelnut skins, compared with ~53% for shells at similar concentrations.

Studies specifically addressing shell-derived extracts have reported lower but still meaningful antioxidant values. Shahidi et al. [36] documented a CUPRAC value of 1.95 μmol TEAC/g for shell extracts, while other investigations have shown that optimized hydrothermal or green extraction conditions can enhance the recovery of antioxidant compounds from the shell matrix [41]. Although the absolute values differ due to methodological variations (solvent, temperature, assay type), the overall trend across studies is consistent: hazelnut skins provide the highest antioxidant activity, while shells despite lower phenolic concentrations still exhibit measurable and functionally relevant antioxidant potential.

The findings of the present study are therefore aligned with the established literature and confirm that both hazelnut skins and shells are promising sources of natural antioxidants. The incorporation of this substance into polymer matrices has the potential to enhance the functional properties of active food packaging systems.

### 3.3. Quantification and Identification of Phenolic Compounds in Hazelnut By-Products by High Pressure Liquid Chromatography (HPLC)

A detailed chromatographic analysis of individual phenolic compounds revealed marked compositional differences between HSkE and HShE (Table 1). HSkE presented a considerably richer profile, particularly in flavanol derivatives. Catechin (438 mg/100 mL) and epigallocatechin gallate (EGCG, 88.84 mg/100 mL) were the predominant compounds in HSkE, whereas their contents in HShE were considerably lower (0.90 and 11.27 mg/100 mL, respectively). These results corroborate earlier findings by Alasalvar et al. [10], who reported hazelnut skin to be an exceptionally rich source of flavonoids, contributing significantly to its antioxidant potential.

In addition to flavanols, HSkE contained higher levels of key phenolic acids. Vanillic acid (29.13 mg/100 mL) and syringic acid (12.71 mg/100 mL) were detected in considerably higher concentrations than in HShE (3.65 mg/100 mL and <0.1 mg/100 mL, respectively). The detection of rutin (16.68 mg/100 mL) exclusively in HSkE and the presence of vanillin only in the skin extract further highlight the qualitative and quantitative diversity of phenolic constituents in the hazelnut skin matrix.

The compositional richness of HSkE compared with HShE is consistent with the well-established anatomical and biochemical differences between hazelnut skin and shell tissues. The former contains higher proportions of metabolically active outer layers where polyphenols accumulate as protective compounds. The higher antioxidant capacity observed for HSkE in CUPRAC and DPPH assays can be explained by this biochemical distribution [42]

In conclusion, the distinct phenolic profiles of HSkE and HShE demonstrate their significance as functional additives in active packaging applications. The presence of flavanols, phenolic acids, and glycosylated flavonoids in HSkE, along with the moderate yet significant phenolic content of HShE, lends support to their potential utilization in biodegradable films designed to enhance antioxidant and antimicrobial performance. Such incorporation may contribute to improved oxidative stability and extended shelf-life of packaged foods, offering a natural and sustainable alternative to synthetic additive [43].

### 3.4. Film Properties

#### 3.4.1. Structural Analysis (FTIR, XRD)

**Figure 2:**
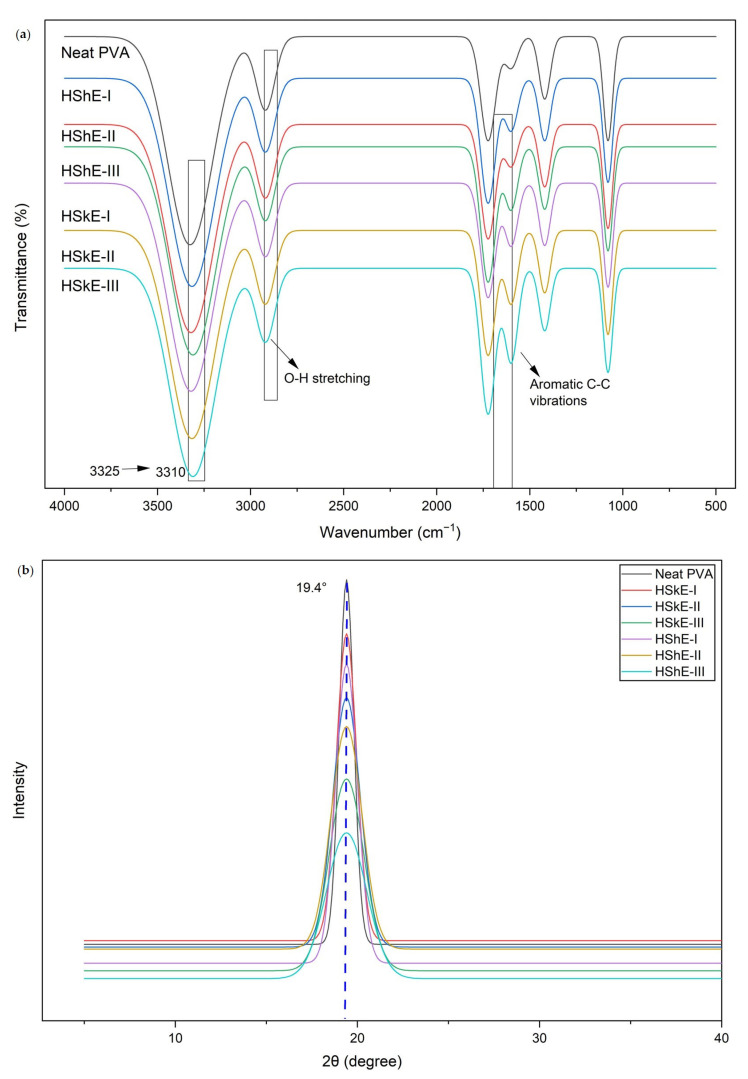
Structural properties of films (**a**) FTIR, (**b**) XRD. HShE: Hazelnut shell extract, HSkE: Hazelnut skin extract (I, II, and III represent films containing 1%, 2%, and 3% (*w*/*v*) of HShE and HSkE, respectively).

Films incorporating hazelnut skin extracts exhibited more pronounced aromatic C=C peaks and slightly greater downshifts of the O–H band, suggestive of enhanced intermolecular interactions, including hydrogen bonding and network restructuring. This phenomenon is likely attributable to the higher concentrations of phenolic compounds, particularly catechins and flavonols, present in hazelnut skins compared to shells [6]. The FTIR spectra of neat PVA and phenolic-enriched composites (HShE and HSkE) reveal spectral changes indicative of molecular interactions between hazelnut-derived polyphenols and the PVA/CMC matrix. Neat PVA/CMC films exhibited characteristic absorption bands, including a broad O–H stretching band at ~3325 cm^−1^, C–H stretching around 2922 cm^−1^, a carbonyl band near 1724 cm^−1^ associated with intrinsic carbonyl groups of the PVA matrix and residual acetate functionalities, and C–O–C and C–O stretching bands at ~1090 and 1048 cm^−1^, which are typical of polysaccharide–polyol systems (Figure 2). The O–H stretching band shifted from 3325 cm^−1^ in the neat film to ~3310 cm^−1^ in films with 3% extract, which may be attributed to changes in hydrogen bonding environments between the hydroxyl groups of polyphenols and PVA/CMC chains [44,45]. This effect was more pronounced in HSkE-based films, consistent with the higher level of flavanols and phenolic acids in the skin extract. In addition, subtle increases in the intensity of aromatic-related bands and slight changes in the carbonyl region suggest the presence of secondary, non-covalent interactions involving phenolic rings and ester groups. These spectral changes support the presence of physical interactions between hazelnut-derived polyphenols and the PVA/CMC matrix, leading to molecular-level network rearrangement without the formation of covalent bonds.

#### 3.4.2. X-ray Diffraction (XRD) Analysis

The X-ray diffraction (XRD) patterns of the PVA/CMC films reinforced with phenolics derived from hazelnut shell and skin at different concentrations (1%, 2%, and 3%) revealed considerable structural differences indicative of molecular interactions and crystallinity changes Figure 2. The neat PVA film exhibited a characteristic diffraction peak at 2θ ≈ 19.4°, typical of PVA-based films due to its semi-crystalline structure. The incorporation of hazelnut phenolic extracts from skin (HSkE) and shell (HShE) resulted in a gradual decrease in peak intensity and an increase in component broadness, particularly at higher extract concentrations (3%). This reduction in crystallinity can be attributed to the interaction of phenolic compounds with the hydroxyl groups of the polymer matrix through hydrogen bonding, which interferes with the regular arrangement of PVA chains and limits crystal growth.

Comparable findings have been consistently reported in the literature. For example, Kang et al. [46] demonstrated that incorporating rose anthocyanins into a PVA/okra mucilage polysaccharide matrix resulted in a marked reduction and broadening of the characteristic crystalline peak of PVA at ~19.5°. This behavior was attributed to the ability of anthocyanin molecules to establish extensive hydrogen bonding with the polymer chains, thereby disrupting the ordered packing of PVA and promoting a transition toward a more amorphous structure. Similarly, Chen et al. [47] observed comparable crystallinity reduction in hydrocolloid-based films enriched with tea polyphenols. Their XRD analyses revealed weakened diffraction peaks, which the authors attributed to strong intermolecular hydrogen bonding between polyphenolic hydroxyl groups and the polymer backbone. Such interactions interfere with crystal domain formation and enhance amorphous character within the composite matrix

This reduction in crystallinity has been widely recognized in the literature as a typical consequence of polyphenol–polymer interactions in biodegradable film systems [46]. Increased amorphous content promotes more uniform dispersion of bioactive phenolic molecules throughout the polymer network, improves chain mobility, and enhances functional performance. As demonstrated in previous studies, films with reduced crystallinity exhibit superior antioxidant capacity and improved UV-light shielding both essential properties for delaying oxidation, preserving sensory attributes, and extending the shelf life of packaged foods [48,49]. In this context, the structural changes observed in the present study align well with established structure–function relationships in polyphenol-reinforced biodegradable films.

#### 3.4.3. Thermal Properties (DSC)

**Figure 3:**
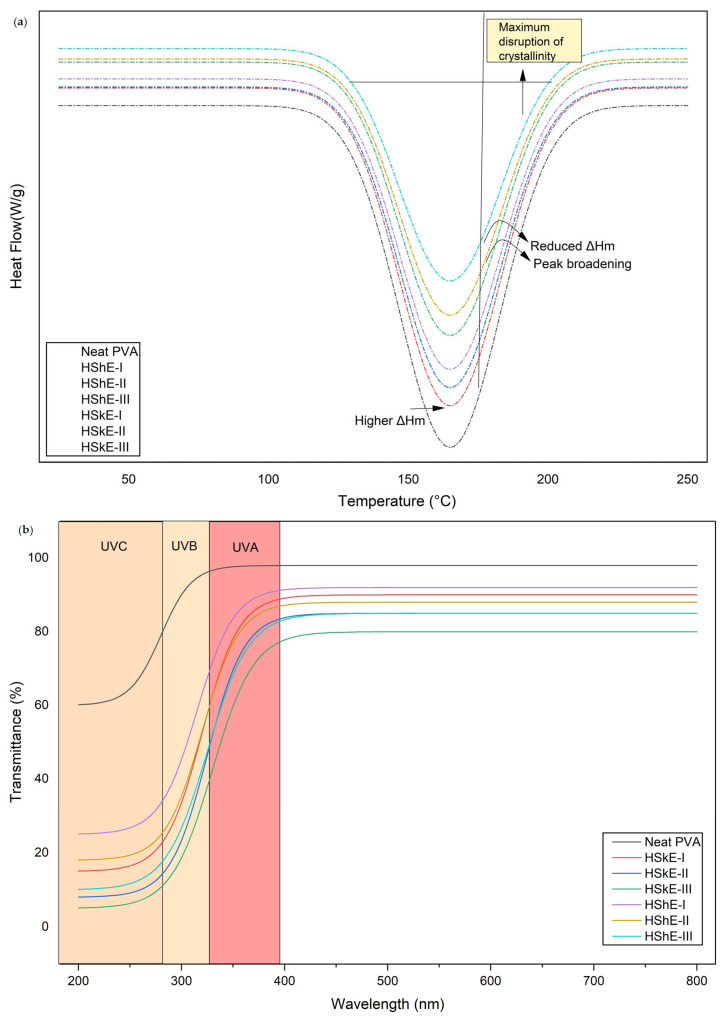
(**a**) Thermal properties (DSC), (**b**) optical properties of films (UV). HShE: Hazelnut shell extract, HSkE: Hazelnut skin extract (I, II, and III represent films containing 1%, 2%, and 3% (*w*/*v*) of HShE and HSkE, respectively).

The DSC thermograms of neat PVA and the composite films containing hazelnut skin and shell extracts displayed a single endothermic peak in the range of 150–165 °C, corresponding to the melting transition (Tm) of PVA crystalline regions (Figure 3). The presence of a single melting peak across all formulations indicates good miscibility between PVA and hazelnut-derived phenolics, with no evidence of phase separation or formation of secondary crystalline phases. This behavior demonstrates that the extracts were uniformly dispersed within the polymer matrix and did not introduce thermally distinct domains [50].

Neat PVA exhibited the most pronounced melting endotherm, with a deep and well-defined peak, reflecting its characteristic semi-crystalline structure. In contrast, all extract-loaded films showed a progressive reduction in peak depth and a noticeable broadening of the endotherm, despite exhibiting only minimal changes in Tm [51]. The nearly unchanged melting temperature suggests that the crystalline lattice of PVA remains structurally intact; however, the significant decrease in melting enthalpy (ΔH_m_) indicates a lower quantity of crystalline domains within the matrix. This trend is consistent with the hypothesis that hazelnut phenolics interfere with polymer chain packing rather than modifying the intrinsic thermal stability of the polymer [52].

This reduction in crystallinity can be attributed to specific hydrogen-bonding interactions between phenolic hydroxyl groups and the hydroxyl-rich PVA/CMC network, which disrupt regular chain alignment and limit crystal growth [50]. This interpretation is strongly supported by the FTIR results, which revealed a downshift and broadening of the O–H stretching band—evidence of intensified inter- and intramolecular hydrogen bonding. Moreover, XRD analysis confirmed a significant decline in diffraction peak intensity at ~19.4°, indicating a shift toward a more amorphous polymer structure. The combined spectroscopic and thermal evidence provides a coherent mechanistic understanding of how hazelnut by-product extracts modulate polymer microstructure.

Among the composite films, the most substantial decrease in peak depth was observed in the 2% extract formulations (HSkE-II and HShE-II), suggesting an optimal interaction level where phenolic molecules are sufficiently concentrated to disrupt crystallinity without causing aggregation or structural discontinuity. At higher concentrations (3%), the effect plateaued, consistent with previously reported interaction saturation phenomena in phenolic–polymer systems [14,53]. Such changes are typically associated with reduced crystalline order and a higher amorphous fraction within the polymer matrix.

The DSC results provide strong quantitative and mechanistic evidence that hazelnut phenolic extracts reorganize the PVA/CMC matrix by reducing crystallinity rather than altering thermal transition temperatures. This reorganized network, which is characterized by greater amorphous content, enhanced chain mobility, and increased free volume correlates directly with improvements in functional performance, including UV-shielding, antioxidant activity, and barrier efficiency. These findings collectively highlight the critical role of phenolic–polymer interactions in shaping the structural and application-relevant thermal behavior of sustainable, bioactive packaging films.

#### 3.4.4. Optical Properties

The UV–Vis transmittance profiles of the films clearly demonstrate that incorporating hazelnut skin and shell extracts significantly enhances the UV-shielding ability of the PVA/CMC matrix (Figure 3). As expected, neat PVA, lacking aromatic chromophores, permitted nearly complete UV transmission, with values exceeding 95% above 350 nm. This confirms its inherently poor UV-blocking ability and its highly transparent semi-crystalline microstructure [23].

In contrast, all extract-loaded films exhibited strong and concentration-dependent UV attenuation. HSkE-based films particularly HSkE-III showed the most pronounced reduction in UV transmittance, remaining below ~10% in the UVC region (200–280 nm) as directly determined from the UV–Vis transmittance spectra and maintaining noticeably lower transmission across the UVB and UVA ranges than both the neat film and HShE formulations. The superior performance of HSkE-containing films directly correlates with their richer phenolic profile, characterized by higher catechin, flavonols, and phenolic acid content. These molecules possess conjugated aromatic structures capable of absorbing high-energy UV photons, which accounts for their strong electronic transitions and corresponding optical barrier effects [6]. HShE films also demonstrated enhanced UV shielding, although to a lesser extent than HSkE films, which is consistent with their lower total phenolic content. For example, UV–Vis transmittance measurements showed that HShE-III films transmitted approximately 20% of incident light at 320 nm, whereas HSkE-III films exhibited lower transmittance values of around 12% at the same wavelength. Importantly, despite the improved UV-blocking performance, all composite films maintained high transparency in the visible region (>70%), indicating that phenolic incorporation does not compromise visual clarity, which is an essential requirement for food packaging applications.

Critically, the enhanced UV-blocking behavior is not solely attributable to the intrinsic absorbance of phenolic compounds. FTIR and XRD analyses provide mechanistic insight into how structural modifications within the polymer matrix influence optical performance. FTIR revealed a downshift and broadening of the O–H stretching band upon phenolic incorporation, indicating intensified hydrogen bonding between polyphenolic hydroxyls and the PVA/CMC matrix. This interaction disrupts the native hydrogen-bond network of PVA, facilitating the formation of a more amorphous structure [23,52].

XRD results further confirmed a reduction in crystallinity, with broadened and weakened peaks at ~19.4° as extract concentration increased. The resulting increase in amorphous content enhances the uniform dispersion of phenolics throughout the matrix. A homogeneous polymer–phenolic network reduces scattering centers and increases the effective interaction between UV photons and aromatic groups, thus synergistically improving UV barrier capacity. These findings are consistent with previously reported polyphenol-reinforced PVA systems [23,52].

Alongside changes in UV behavior, hazelnut phenolics also induced measurable shifts in film color. Neat PVA exhibited a high L* value (85.05), indicative of a bright, transparent matrix. Increasing extract concentration produced slight reductions in L*, particularly in HSkE-II and HSkE-III, reflecting mild darkening due to natural pigment incorporation. Elevated a* and b* values across all films signaled a shift towards red and yellow hues, commonly associated with phenolic chromophores. Total color difference (ΔE) values exceeded 2 for all enriched films, with HSkE-III showing the highest deviation (ΔE = 6.24). Importantly, these moderate color modifications do not negatively impact optical appearance but instead provide visual confirmation of active compound incorporation. These color changes are therefore associated with the incorporation of phenolic compounds and remain within acceptable limits for active food packaging applications [46,50].

The evidence from optical, spectroscopic and crystallographic analyses demonstrates that hazelnut-derived phenolics enhance the UV-blocking efficiency of the PVA/CMC network through intrinsic absorption, as well as structurally reorganizing it to support improved dispersion and reduced crystallinity. The synergistic molecular-level interactions result in films that exhibit a combination of properties, namely ultraviolet (UV) protection, visual transparency, structural modification, and functional activity. This significant enhancement in their suitability for sustainable active packaging systems is a notable advancement.

#### 3.4.5. Mechanical Properties

**Figure 4:**
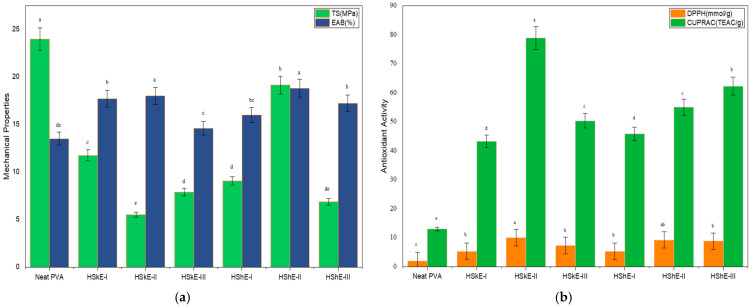
(**a**) Mechanical properties and (**b**) antioxidant activity of films. HShE: Hazelnut shell extract, HSkE: Hazelnut skin extract (I, II, and III represents films containing 1%, 2%, and 3% (*w*/*v*) of HShE and HSkE, respectively).  Different lowercase letters (a–e) indicate significant differences among samples (*p* < 0.05).

The mechanical performance of biodegradable packaging films is closely tied to their molecular organization, hydrogen-bonding network, and the degree of crystallinity within the polymer matrix [52]. In the present study, neat PVA film (control) exhibited the highest tensile strength (TS) of 23.99 MPa, a result consistent with its semi-crystalline morphology and the presence of dense inter-chain hydrogen bonding that stabilizes the polymer network (Figure 4).

With the incorporation of hazelnut-derived phenolic extracts, a statistically significant (*p* < 0.05) and concentration-dependent reduction in TS was observed, with the most pronounced decrease occurring in HShE-III (5.53 MPa). This phenomenon of mechanical weakening can be attributed to several molecular-level phenomena. FTIR analysis indicated an intensification of hydrogen bonding between phenolic hydroxyl groups and the PVA/CMC matrix, this was evidenced by a downshift of the O–H stretching band. While this interaction reinforces certain localized regions, it simultaneously disrupts native PVA–PVA and PVA–CMC hydrogen bonds, creating microstructural discontinuities and reducing cohesive energy density. Additionally, XRD results supported a decrease in crystallinity with increasing extract concentration, showing broadened and weakened diffraction peaks. As polymer crystallinity is a key determinant of tensile strength, this reduction provides a mechanistic explanation for the observed TS decline [12,50].

Similar behaviors have been documented in other polyphenol-reinforced films, where aromatic compounds or essential oils interfere with the orderly packing of polymer chains, leading to diminished mechanical resistance [54]. The findings of this study therefore align with literature, confirming that phenolic additives introduce amorphous character and structural heterogeneity that weaken the load-bearing capability of the films [12,52].

Conversely, elongation at break (EAB) increased upon extract incorporation, reaching 18.84 ± 0.37% for HSkE-II and 18.01 ± 0.42% for HShE-II, compared with 16.01 ± 0.40^b^% for neat PVA film. This enhancement in flexibility suggests a plasticizing effect of the extracts. Phenolic molecules, which contain multiple hydroxyl groups, can position themselves between polymer chains, increasing free volume and chain mobility while serving as secondary “soft” interaction sites [55]. The FTIR-indicated hydrogen bonding between phenolic and the polymer matrix contributes to this increased mobility by loosening the tight hydrogen-bonding network normally present in neat PVA/CMC systems. Furthermore, the reduction in crystallinity observed in XRD promotes a more amorphous matrix, which typically correlates with improved ductility and reduced brittleness.

However, at the highest extract concentration (3%), EAB showed a slight decline. This behavior may originate from localized aggregation or phase separation of phenolics, which creates rigid microdomains that limit polymer chain movement. Such non-uniform dispersion has been noted in other phenolic-rich films and marks the threshold beyond which excessive additive loading becomes detrimental to mechanical flexibility [56].

Collectively, these results highlight the role of hazelnut-derived phenolics in modulating the mechanical performance of PVA/CMC films. While tensile strength decreases due to structural disruption and reduced crystallinity, ductility improves as the amorphous content and chain mobility increase. This combination of lower stiffness and higher flexibility is desirable for applications that prioritize conformability and resistance to deformation rather than high tensile load such as wrapping soft fruits, fresh-cut produce, or irregularly shaped food items. Integrating mechanical results with FTIR, XRD, and optical findings therefore demonstrates that the molecular interactions introduced by phenolics not only modify mechanical response but also contribute to enhanced functional behavior within the broader packaging context.

#### 3.4.6. Barrier Properties (WVP, OP)

Barrier properties are a critical determinant of food packaging performance, as they directly influence product stability, oxidative deterioration, microbial susceptibility, and overall shelf-life. In the context of the properties of the materials under consideration, resistance to water vapor and oxygen transmission is of particular significance. The underlying rationale for this is that both moisture uptake and oxidative reactions can accelerate texture degradation, pigment loss, rancidity, and nutrient depletion during storage [57]. The neat PVA film demonstrated the highest WVP value (10.4 × 10^−2^ g·mm/m^2^·h·kPa), consistent with its hydrophilic nature and high density of hydroxyl groups capable of interacting with water molecules. This strong affinity for moisture facilitates rapid diffusion through the polymer network, resulting in poor moisture barrier performance (Table 2).

The incorporation of hazelnut phenolic extracts (HSkE and HShE) led to a significant (*p* < 0.05) reduction in WVP, with the smallest reduction observed in HShE-I (8.8 × 10^−2^ g·mm·m^−2^·h^−1^·kPa^−1^). This reduction can be attributed to several synergistic structural effects. FTIR results revealed intensified hydrogen bonding between polyphenolic hydroxyl groups and the PVA/CMC matrix, suggesting that phenolics occupy functional sites previously available for water–polymer interactions. This reduces the number of hydrophilic free hydroxyl groups available to form hydrogen bonds with water, thereby decreasing moisture uptake within the film [12].

Furthermore, XRD analysis demonstrated a reduction in crystallinity upon phenolic incorporation, resulting in a more amorphous yet structurally compact matrix. While increased amorphous character typically enhances permeability, in this case the phenolics appear to fill microvoids and reduce porosity, thus impeding water vapor diffusion. This “matrix densification effect” is particularly effective at low extract concentrations (1–2%), where phenolics disperse homogeneously and establish extensive hydrogen bonding. However, at 3% loading, WVP did not continue to decrease, likely due to local phenolic aggregation or partial phase separation. Such microstructural imperfections act as diffusion pathways, counteracting the barrier-enhancing effect and supporting the polymer–extract interaction plateau described in previous studies [50,58]

Oxygen permeability (OP) exhibited an even more pronounced improvement upon the incorporation of hazelnut phenolics. Neat PVA displayed an OP of 0.048 cm^3^·mm·m^−2^·day^−1^·atm^−1^, whereas HSkE films achieved reductions of 35.4%, 52.1%, and 68.8% at 1%, 2%, and 3% concentrations, respectively. A similar concentration-dependent decrease was observed for HShE films (27.1%, 47.9%, and 66.7%). This systematic enhancement in oxygen barrier performance underscores the strong influence of phenolic-polymer interactions [59].

Mechanistically, the formation of hydrogen bonds between the phenolic hydroxyl groups and the hydroxyl-rich PVA/CMC matrix increases cohesive energy density and restricts chain mobility. This reduces the free volume and slows the diffusion of oxygen molecules. Additionally, phenolics partially occupy amorphous microvoids and create tortuous diffusion pathways, forcing oxygen molecules to navigate longer, less direct routes through the polymer matrix. The resulting structural compaction significantly impedes oxygen permeation, aligning with observations made in other natural phenolic-enriched packaging films [53,59].

The WVP and OP results demonstrate a discernible structure–property relationship. FTIR analysis confirmed enhanced hydrogen bonding, XRD analysis confirmed reduced crystallinity and increased amorphous density, and barrier tests demonstrated that these molecular rearrangements yield films with superior resistance to moisture and oxygen transmission. The findings are of particular significance in the context of food packaging, where oxygen-sensitive foods (fresh-cut fruits, nuts, meats) benefit greatly from materials that retard oxidation and moisture exchange. It can thus be concluded that hazelnut-derived phenolics represent a promising multifunctional reinforcement capable of improving barrier integrity while simultaneously contributing antioxidant capacity.

#### 3.4.7. Antioxidant Activity (AA)

The AA of the biodegradable films was evaluated using DPPH and CUPRAC assays, and the results are shown in Figure 4. The neat PVA exhibited the lowest antioxidant activity, with DPPH and CUPRAC values of 2.01 and 13.04, respectively. This behavior is consistent with its chemical structure, which lacks aromatic rings, phenolic hydroxyl groups, or conjugated systems capable of participating in electron transfer or hydrogen atom donation [60]

Incorporation of hazelnut skin and shell extracts resulted in a marked enhancement of antioxidant activity, demonstrating the strong free radical–scavenging potential of phenolic-enriched films. Among the HSkE-based formulations, HSkE-II displayed the highest activity (DPPH: 10.02 mmol/g; CUPRAC: 78.9 TEAC/g), indicating that this concentration provides an optimal balance between phenolic availability and molecular dispersion within the polymer network. HShE-based films also exhibited substantially greater antioxidant activity than neat PVA, consistent with the presence of phenolic acids (e.g., gallic, syringic, protocatechuic acids) and flavanols detected in the extract characterization [6,41].

Importantly, the enhanced antioxidant behavior can be mechanistically linked to structural interactions identified in FTIR and XRD analyses. FTIR revealed intensified hydrogen bonding between phenolic hydroxyl groups and the PVA/CMC matrix, suggesting that phenolics are well integrated into the polymer network. Such interactions not only stabilize phenolics within the film but also facilitate their controlled release at the film surface, where radical scavenging reactions occur. XRD further showed that increasing extract concentration reduced crystallinity and increased the amorphous fraction, creating a more open polymeric structure that enhances molecular mobility and allows greater accessibility of phenolics to reactive oxygen species.

This relationship between structural reorganization and antioxidant function is consistent with previously reported findings in polyphenol-rich active films [51]. In amorphous matrices, phenolics are more uniformly distributed and less sterically hindered, enabling stronger electron-donating capacity and more efficient radical neutralization. The slightly lower activity observed at the highest extract concentration (3%) in some formulations may be explained by localized phenolic aggregation, which reduces surface availability and limits effective radical interaction, a behavior commonly reported in polyphenol-loaded polymer systems.

The results obtained demonstrate that phenolic compounds derived from hazelnut skin and shell function as effective natural antioxidants when incorporated into PVA/CMC matrices. The enhanced antioxidant performance is closely associated with the structural modifications observed through Fourier-transform infrared spectroscopy (FTIR) and X-ray diffraction (XRD) analyses. The formation of additional hydrogen bonds between phenolic hydroxyl groups and the polymer network has been shown to alter the native hydrogen-bonding environment of PVA/CMC. In addition, a reduction in crystallinity has been observed, which has the effect of promoting a more amorphous and permissive matrix structure. The combination of phenolics within the film has been shown to enhance the uniform distribution and molecular accessibility of these compounds, thereby increasing their ability to participate in radical-scavenging and electron-transfer reactions [50,52].

The synergistic interaction between these structural and functional changes highlights the potential of hazelnut by-product extracts to serve as bioactive components in active packaging materials designed to mitigate oxidative degradation and extend the shelf life of perishable foods.

#### 3.4.8. Biodegradation Analysis

**Figure 5:**
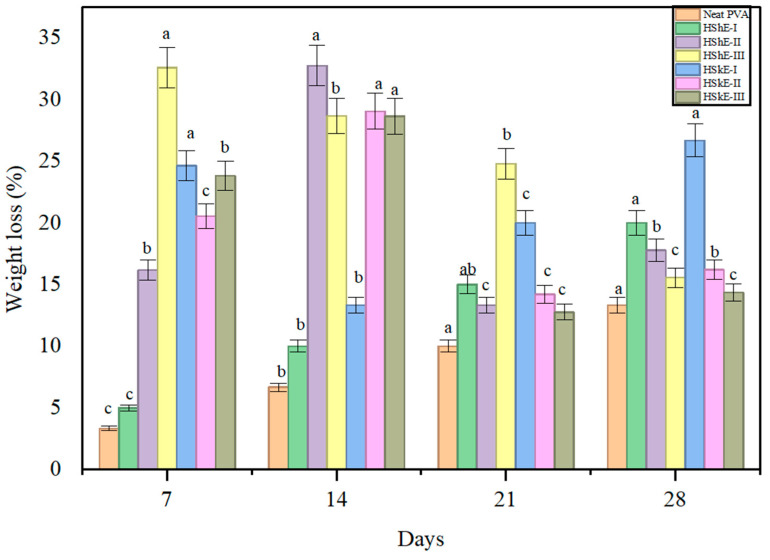
Biodegradation Analysis of films. HShE: Hazelnut shell extract, HSkE: Hazelnut skin extract (I, II, and III represents films containing 1%, 2%, and 3% (*w*/*v*) of HShE and HSkE, respectively). Different lowercase letters (a–c) indicate significant differences among samples (*p* < 0.05).

The biodegradation behavior of packaging materials in soil is a critical parameter for assessing their environmental impact and long-term sustainability. Soil degradation rates are influenced by multiple factors, including temperature, moisture, microbial diversity, and, importantly, the chemical composition and structural characteristics of the films themselves [61]. In this study, biodegradability was assessed through the gravimetric measurement of weight loss over a 28-day soil burial test (Figure 5).

Neat PVA exhibited a weight loss of 13.33% at the end of the test period, consistent with its partially biodegradable nature and semi-crystalline structure, which limits microbial accessibility. In contrast, films containing hazelnut shell extract (HShE) displayed higher degradation values (15.56–20.00%), while HSkE-containing films showed the most pronounced biodegradation, reaching up to 26.67%. These results clearly indicate that the incorporation of phenolic-rich extracts enhances the biodegradability of the composite films, particularly at lower concentrations [62].

The observed enhancement in degradation can be attributed to several molecular-level mechanisms. First, FTIR analysis demonstrated the formation of additional hydrogen bonds between phenolics and the PVA/CMC matrix, which alters the polymeric network and increases the availability of hydrophilic functional groups. Increased hydrophilicity facilitates water penetration into the film, accelerating the initial swelling and subsequent microbial colonization. Second, XRD analysis showed that phenolic incorporation reduces crystallinity and increases amorphous content regions that are more susceptible to enzymatic and microbial attack, as amorphous domains degrade more readily than tightly packed crystalline structures.

Interestingly, films with higher extract concentrations (HShE-III and HSkE-III) exhibited slightly reduced biodegradation compared to their lower-concentration counterparts. This behavior may be linked to the intrinsic antimicrobial and antioxidant properties of phenolic compounds, which, at higher loadings, may inhibit soil microbial activity locally. Additionally, excess phenolic content may lead to microstructural densification or aggregation within the matrix, limiting the accessibility of microbes and slowing degradation. This concentration-dependent trend reflects the need for an optimal extract loading that balances structural reinforcement, functional performance, and environmental degradation [63,64].

These findings align with the results of Singhaboot et al. [55] who reported enhanced biodegradation in PVA-based films reinforced with biogenic additives, with degradation rates increasing by up to 36.57% within 14 days when amorphous content and hydrophilicity were elevated. Collectively, the present study demonstrates that hazelnut by-product extracts not only contribute functional antioxidant and barrier properties but also significantly promote biodegradability when incorporated at suitable concentrations, supporting their potential in the development of sustainable active packaging materials.

### 3.5. Applications of the Films on Fresh Fruits and Chicken

#### 3.5.1. Appearance and Color

**Figure 6:**
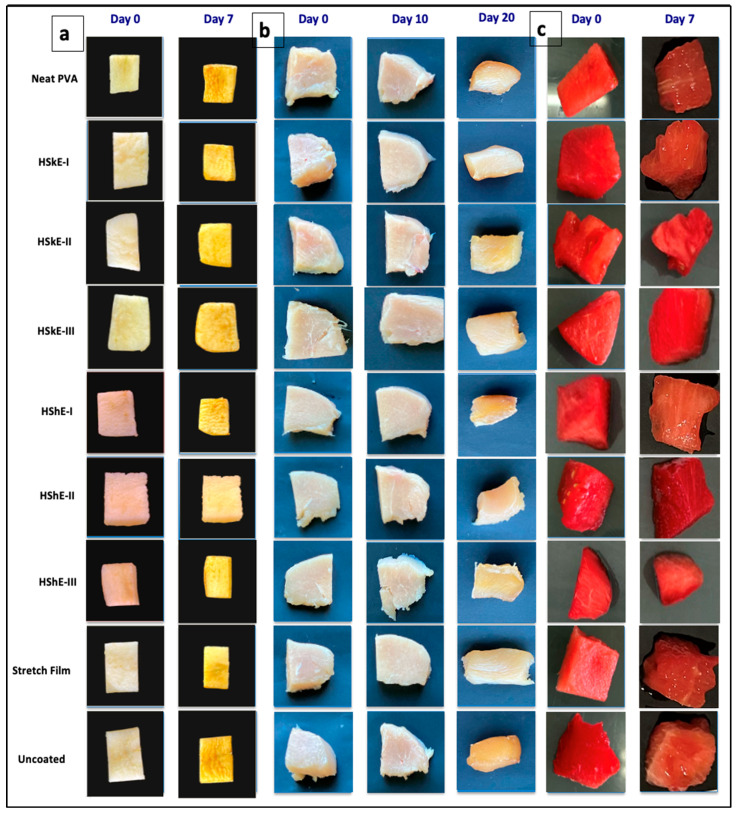
Visual appearance of (**a**) fresh-cut apples, (**b**) chicken and (**c**) watermelons during storage.

The suitability of PVA–HShE and PVA–HSkE composite films for food preservation was assessed using fresh-cut apples, watermelon and chicken breast stored under refrigeration. Photographic documentation on day 0 and day 7 (Figure 6) revealed clear differences among treatments. It was evident that both uncoated samples and those coated with neat PVA exhibited significant deterioration by day 7, characterized by browning, tissue softening, and moisture loss. In contrast, films fortified with hazelnut phenolic extracts, particularly at a concentration of 2%, retained the appearance and firmness of the fruit samples. This suggests that there was an improvement in oxidative stability during storage.

Colorimetric measurements supported these observations. Fresh-cut apples typically undergo browning characterized by an increase in a* values due to enzymatic oxidation [65]. In fresh-cut apples, uncoated samples exhibited a pronounced increase in a* values from 1.0 on day 1 to 8.0 on day 7. Samples coated with HShE-II and HSkE-II exhibited significantly smaller changes in both parameters compared with controls, indicating effective inhibition of color degradation. Similarly, watermelon slices coated with phenolic-enriched films showed minimal changes in lycopene-associated color attributes, consistent with reduced pigment oxidation [66]. In the case of watermelon slices, a substantial decrease in a* values from 26.6 to 21.8 was observed in uncoated samples over the 7-day storage period. Conversely, phenolic-enriched films effectively preserved color. In particular, HSkE-II and HShE-II maintained a* values of 25.6 and 25.8 on day 7, compared with 24.6 and 24.5 for stretch and neat PVA films, respectively. The most pronounced color deterioration occurred in uncoated samples, while the phenolic-containing films, especially at 2%, preserved the visual quality more effectively.

**Figure 7:**
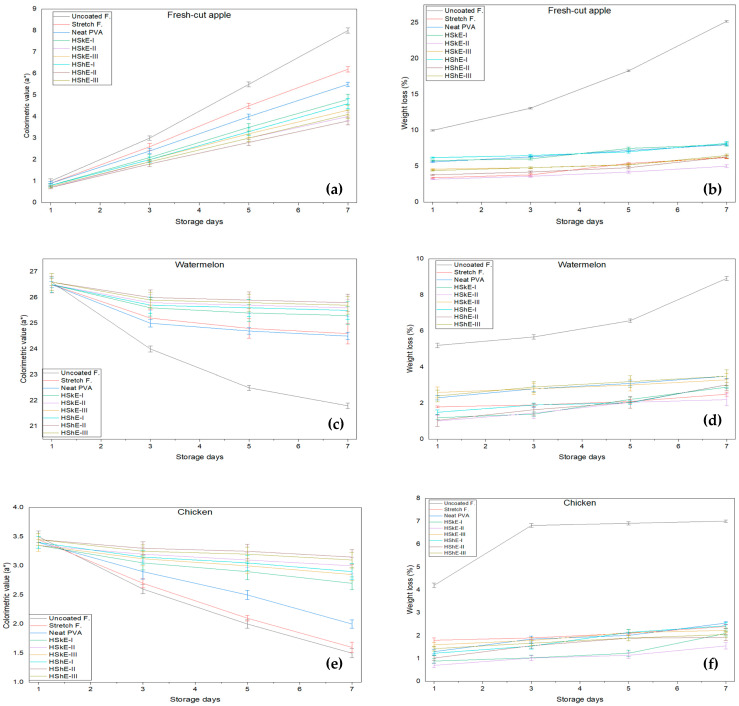
The color change and weight loss of fresh-cut apple, fresh watermelon and chicken in storage days.HShE: Hazelnut shell extract, HSkE: Hazelnut skin extract (I, II, and III represents films containing 1%, 2%, and 3% (*w*/*v*) of HShE and HSkE, respectively).(**a**) Colorimetric value (a*) and (**b**) weight loss (%) of fresh-cut apples, (**c**) Colorimetric value (a*) and (**d**) weight loss (%) of watermelon, (**e**) Colorimetric value (a*) and (**f**) weight loss (%) of chicken.

In chicken samples, the decline in a* values over the storage period reflected the expected oxidation of myoglobin. However, the rate of color loss differed significantly among treatments (*p* < 0.05). The most rapid reduction in redness during storage was exhibited by uncoated and neat PVA-coated samples, with a* values in uncoated samples decreasing from 3.5 to 1.5. In contrast, coatings incorporating hazelnut-derived phenolics, namely HShE-II and HShE-III, exhibited higher a* values throughout the storage period, reaching 3.15 and 3.10, respectively, on day 7 (Figure 7). This enhanced color stability is likely associated with the antioxidant properties of the extracts and the reduced oxygen permeability observed in the composite films, both of which help limit oxidative reactions in muscle tissues. Similar protective effects have been reported in meat systems treated with phenolic-containing biopolymer coatings [67].

The results demonstrate that the incorporation of hazelnut skin and shell extracts into PVA/CMC matrices enhances the visual quality and color stability of both plant- and animal-based foods during refrigerated storage. These findings underscore the potential of hazelnut-derived phenolics as functional components for active, biodegradable packaging systems.

#### 3.5.2. Weight Loss

Weight loss is a critical indicator of freshness in high-moisture fruits and meat, as it reflects transpiration, respiration, and surface dehydration during storage. Over the 7-day refrigerated period, all uncoated samples showed the greatest loss in mass, reaching 8.92% for watermelon, 25.2% for apple, and 7.01% for chicken (Figure 7). This behavior is consistent with unrestricted moisture transfer between the product and the surrounding environment, emphasizing the inadequacy of unprotected storage for retaining food quality [15,68].

In contrast, films containing hazelnut shell or skin phenolic extracts significantly reduced weight loss compared with neat PVA and uncoated controls (*p* < 0.05). For watermelon, HSkE-II (2.2%) and HShE-II (3.02%) provided the strongest moisture retention, performing comparably to or better than commercial stretch film (2.5%). A similar pattern was observed in apples, where HSkE-II (5.01%) and HShE-II (6.3%) again demonstrated superior barrier performance by restricting dehydration more effectively than neat PVA or 1% extract films.

The most pronounced effect was observed in chicken samples, where HSkE-II limited weight loss to only 1.55%, outperforming both stretch film (2.4%) and all other tested formulations. Coatings such as HSkE-I (2.1%) and HShE-II (1.92%) also conferred substantial moisture-protective benefits, confirming that phenolic incorporation strengthens the functional capacity of PVA/CMC matrices. The improved performance of HSkE-II across all food types likely reflects the combined influence of its enhanced network structure, reduced water vapor permeability, and the hydrophilic–hydrophobic balance conferred by the phenolic composition of the extract [52].

The findings demonstrate that the incorporation of hazelnut skin and shell extracts into biodegradable films can effectively mitigate storage-induced weight loss in both fruits and chicken. In several cases, their performance is comparable to that of conventional stretch film, highlighting their potential as a sustainable alternative for packaging moisture-sensitive foods.

#### 3.5.3. Microbial Evaluation

The antimicrobial performance of the developed films was assessed by monitoring *S. typhimurium* growth in chicken samples during refrigerated storage (Table 3). The uncoated samples showed rapid microbial proliferation throughout the 15-day period, reaching values above the acceptable microbial limit by the end of storage. Commercial stretch film displayed a similar trend and did not provide any measurable antimicrobial protection.

Neat PVA films resulted in a slight reduction in bacterial growth compared with the uncoated group, which may be attributed to limited oxygen and moisture regulation. However, the inhibitory effect remained modest. In contrast, films incorporating hazelnut phenolic extracts particularly those containing hazelnut skin extract demonstrated a more pronounced suppression of *S. typhimurium* during storage (*p* < 0.05). Samples coated with these films consistently exhibited lower bacterial counts at each sampling point. This behavior can be associated with the higher phenolic content of the skin extract, which includes compounds known to interfere with microbial membrane integrity and oxidative balance [14,41]. Films containing hazelnut shell extract also reduced microbial growth relative to neat PVA and uncoated controls, although the extent of inhibition was generally less marked, in line with the lower phenolic concentration of the shell extract.

The results obtained demonstrate that the incorporation of hazelnut by-product phenolics into PVA/CMC matrices has a significant effect on the antimicrobial performance of the resulting films. The observed inhibitory effect suggests that such films may contribute to improved microbial stability of poultry products during refrigerated storage, supporting their potential application as natural active packaging materials.

#### 3.5.4. Lipid Oxidation

**Figure 8:**
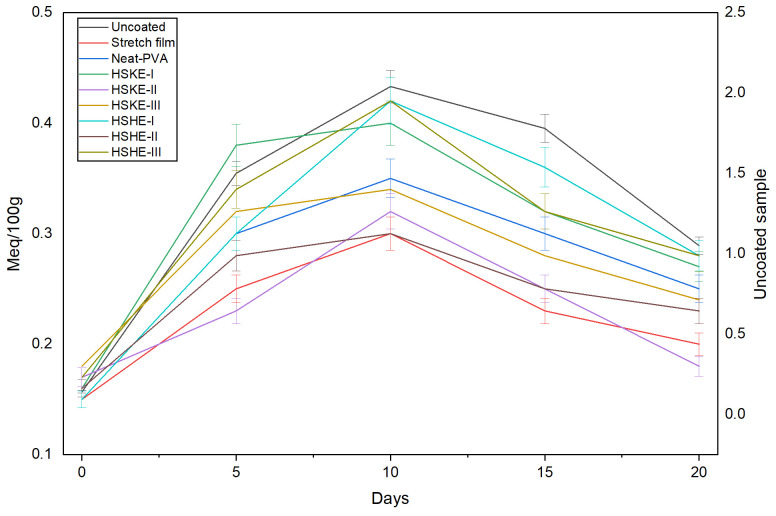
The peroxide value of chicken sample during storage.

Lipid oxidation in chicken samples was monitored through peroxide value (PV) measurements during refrigerated storage (Figure 8). The uncoated control exhibited the highest PV throughout the storage period, reflecting its susceptibility to oxidative deterioration in the absence of protective packaging. Both stretch film and neat PVA provided partial protection, delaying but not preventing oxidation, as indicated by the gradual PV increase observed up to day 10.

Incorporation of hazelnut phenolic extracts considerably improved oxidative stability. Films containing hazelnut skin (HSkE) and shell extracts (HShE) consistently yielded lower PVs compared to other treatments (*p* < 0.05). Notably, HSkE-II and HShE-II maintained the lowest PV by day 20 (0.18 and 0.23 meqV/100 g, respectively), suggesting that phenolic constituents effectively scavenged primary oxidation products and stabilized lipids during storage. These findings are in line with reports showing that phenolic-enriched coatings can inhibit peroxide formation in poultry stored under refrigeration [67]. Differences in PV trends compared with studies reporting higher values may be attributed to variations in storage temperature and oxidative stress conditions [30].

The findings of the present study demonstrate that the utilization of biodegradable PVA films fortified with hazelnut by-product extracts provides efficacious oxidative protection, with the potential to retard lipid degradation in comparison to both conventional polyethylene stretch film and unfortified PVA. The enhanced stability exhibited by phenolic-based active films under chilled storage conditions signifies their considerable potential to prolong the shelf life of chicken products.

## 4. Conclusions

This study presents a sustainable and value-added approach to transforming hazelnut shell and skin two abundant agro-industrial by-products into functional components for biodegradable PVA/CMC packaging films. Phenolic-rich extracts were efficiently recovered and incorporated into film matrices using the green, solvent-free technique of PHWE, demonstrating a practical route for upcycling food waste into high-performance packaging materials. This aligns directly with current sustainability goals aimed at reducing reliance on petroleum-based plastics, lowering environmental burden, and promoting circular bioeconomy practices.

The incorporation of hazelnut phenolics significantly improved the films’ UV-shielding ability, antioxidant capacity, and gas barrier performance, while maintaining desirable transparency and flexibility. Structural and thermal analyses confirmed that these enhancements were derived from strengthened hydrogen bonding, reduced crystallinity, and the formation of a denser polymer network. Importantly, the application studies on fresh-cut apples, watermelon, and chicken verified the practical benefits of these films, including reduced weight loss, delayed color degradation, lower lipid oxidation, and inhibited microbial growth compared with neat PVA and commercial stretch film.

This study provides substantial evidence that hazelnut by-products can be effectively valorized into active packaging materials with multifunctional protective properties. The incorporation of waste-derived phenolics into biodegradable matrices has resulted in the development of films that offer an environmentally responsible alternative to conventional plastics. Concurrently, these films contribute to food quality preservation and waste reduction. These findings underscore the potential of agro-industrial by-products as renewable resources for the development of next-generation sustainable packaging solutions. It is recommended that future studies concentrate on the scaling up of production and the migration behavior of bioactive components to support industrial application and food safety compliance.

## Figures and Tables

**Table 1:** Total phenolic content (TPC), total antioxidant activity (TAA), and phenolic compounds of HShE and HSkE.

	HShE	HSkE
TPC (mg GAE/g dw) (Folin–Ciocalteu)	25.44 ± 1.42 ^a^	83.68 ± 2.69 ^b^
TAA (mg TEAC/g dw) (CUPRAC)	331.23 ± 10.5 ^a^	638.47 ± 9.8 ^b^
Phenolic Compound (mg/100 mL)		
Gallic Acid	6.89	36.05
Catechin	0.90	438
Epigallocatechin gallate (EGCG)	11.27	88.84
Vanillic Acid	3.65	29.13
Syringic Acid	<0.1	12.71
Vanillin	6.32	ND
Rutin	ND	16.68
Trans-Cinnamic Acid	0.13	<0.1

HShE: Hazelnut shell extract, HSkE: Hazelnut skin extract, GAE: Gallic acid equivalent, TEAC: Trolox equivalent. Different letters in the same column indicate significant differences in mean (*p* < 0.05).

**Table 2:** Results of color, thickness and WVP and OP measurements of HShE and HSkE.

Sample Name	L*	a*	b*	ΔE	Thickness (mm)	WVP (g·mm/m^2^·h·kPa)	OP (cm^3^mm/m^2^·day·atm)
Neat PVA	85.05 ^b^	0.98 ^e^	−1.39 ^d^	1.10 ^d^	0.045 ^d^	10.4 × 10^−2 a^	0.048 ^a^
HSkE-I	85.23 ^b^	1.007 ^d^	1.78 ^b^	3.17 ^c^	0.079 ^b^	9.88 × 10^−2 b^	0.031 ^b^
HSkE-II	84.22 ^d^	1.007 ^d^	1.28 ^b^	4.03 ^b^	0.084 ^a^	9.48 × 10^−2 b^	0.023 ^c^
HSkE-III	83.28 ^e^	1.25 ^c^	1.007 ^c^	6.24 ^a^	0.090 ^a^	11.5 × 10^−2 a^	0.015 ^d^
HShE-I	86.29 ^a^	1.64 ^a^	2.79 ^a^	5.52 ^a^	0.069 ^c^	8.8 × 10^−2 c^	0.035 ^b^
HShE-II	86.37 ^a^	1.68 ^a^	2.30 ^a^	5.53 ^a^	0.076 ^b^	9.5 × 10^−2 b^	0.025 ^c^
HShE-III	84.84 ^c^	1.51 ^b^	2.27 ^a^	5.12 ^a^	0.084 ^a^	10.7 × 10^−2 a^	0.016 ^d^

HShE: Hazelnut shell extract, HSkE: Hazelnut skin extract, L* (measurable in terms of white to black intensity), a* (measurable in terms of red and green intensity) and b* (measurable in terms of yellow and blue intensity) of the Hunter system, WVP: Water vapor permeability. Different lowercase letters (a–d) indicate significant differences among samples (*p* < 0.05).

**Table 3:** *S. typhimurium* load of chicken samples during fifteen days of storage (CFU/g).

Sample Name	Storage Time (Days)			
	0	5	10	15
Uncoated	6.21 ± 0.1 ^a^	7.19 ± 0.2 ^a^	8.23 ± 0.8 ^a^	107.23 ± 9.8 ^a^
Stretch film	6.25 ± 0.3 ^a^	4.23 ± 0.25 ^a^	4.52 ± 0.23 ^a^	67.02 ± 0.2 ^b^
Neat PVA	6.24 ± 0.2 ^a^	6.21 ± 0.12 ^a^	7.01 ± 0.23 ^c^	55.12 ± 1.8 ^c^
HShE-I	6.32 ± 0.32 ^a^	4.79 ± 0.18 ^a^	5.02 ± 1.8 ^d^	47.19 ± 0.8 ^d^
HShE-II	6.28 ± 0.23 ^a^	4.01 ± 0.13 ^a^	5.19 ± 0.28 ^d^	27.9 ± 0.38 ^d^
HShE-III	6.2 ± 0.21 ^a^	5.53 ± 0.23 ^a^	6.07 ± 0.8 ^e^	37.12 ± 0.5 ^e^
HSkE-I	6.25 ± 0.18 ^a^	5.32 ± 0.13 ^a^	7.22 ± 0.23 ^c^	53.17 ± 0.3 ^c^
HSkE-II	6.29 ± 0.31 ^a^	4.21 ± 0.28 ^a^	5.24 ± 0.21 ^cd^	45.23 ± 2.1 ^cd^
HSkE-III	6.19 ± 0.27 ^a^	5.29 ± 0.28 ^a^	6.02 ± 0.4 ^d^	53.2 ± 0.8 ^d^

HShE: Hazelnut shell extract, HSkE: Hazelnut skin extract. Different letters in the same column indicate significant differences in mean (*p* < 0.05).

## Data Availability

The original contributions presented in the study are included in the article; further inquiries can be directed to the corresponding author.
